# Influence of different restorative techniques on marginal seal of class II composite restorations

**DOI:** 10.1590/S1678-77572010000100008

**Published:** 2010

**Authors:** Sinval Adalberto RODRIGUES JUNIOR, Lúcio Fernando da Silva PIN, Giovanna MACHADO, Álvaro DELLA BONA, Flávio Fernando DEMARCO

**Affiliations:** 1 DDS, MS, PhD, Substitute Professor, Department of Semiology and Clinics, Dental School, Federal University of Pelotas, Pelotas, RS, Brazil.; 2 DDS, Dentist of the Brazilian Army, Palmas do Tocantins, TO, Brazil.; 3 PhD, Research Scientist, Geology Department, Federal University of Rio Grande do Sul, Porto Alegre, RS, Brazil.; 4 DDS, MS, PhD, Senior Professor, Department of Restorative Dentistry, Dental School, University of Passo Fundo, Passo Fundo, RS, Brazil.; 5 DDS, PhD, Professor, Department of Restorative Dentistry, Dental School, Federal University of Pelotas, Pelotas, RS, Brazil.

**Keywords:** Restoration, Composite resin, Open sandwich technique, Amalgam, Microleakage

## Abstract

**Objective:**

To evaluate the gingival marginal seal in class II composite restorations using different restorative techniques.

**Material and Methods:**

Class II box cavities were prepared in both proximal faces of 32 sound human third molars with gingival margins located in either enamel or dentin/cementum. Restorations were performed as follows: G1 (control): composite, conventional light curing technique; G2: composite, soft-start technique; G3: amalgam/composite association (amalcomp); and G4: resin-modified glass ionomer cement/ composite, open sandwich technique. The restored specimens were thermocycled. Epoxy resin replicas were made and coated for scanning electron microscopy examination. For microleakage evaluation, teeth were coated with nail polish and immersed in dye solution. Teeth were cut in 3 slices and dye penetration was recorded (mm), digitized and analyzed with Image Tool software. Microleakage data were analyzed statistically by non-parametric Kruskal-Wallis and Mann-Whitney tests.

**Results:**

Leakage in enamel was lower than in dentin (p<0.001). G2 exhibited the lowest leakage values (p<0.05) in enamel margins, with no differences between the other groups. In dentin margins, groups G1 and G2 had similar behavior and both showed less leakage (p<0.05) than groups G3 and G4. SEM micrographs revealed different marginal adaptation patterns for the different techniques and for the different substrates.

**Conclusion:**

The soft-start technique showed no leakage in enamel margins and produced similar values to those of the conventional (control) technique for dentin margins.

## INTRODUCTION

The gingival margins of class II restorations are critical to the bonding process because of minimal or total absence of enamel. The composite resin polymerization shrinkage can produce the breakdown of the adhesive bonds. As a consequence, marginal gaps may occur and induce tooth sensitivity and pulpal damages. In addition, the main reason for failure of direct composite restorations has been related to the secondary caries^12^, which still has been associated to both, poor marginal adaptation and sealing^14^.

The open sandwich technique, using glass ionomer cement (GIC) and composite resin, has been suggested as a better option to the conventional composite resin technique^2,11^. The GIC is capable of chemically reacting with calcium ions present in the tooth structure creating a bond between them, providing a better and long-lasting sealing^5^. The addition of resinous particles to increase the mechanical properties and decrease the solubility made the exposure of the RMGIC to the oral environment less critical than its precursor (GIC)^5^.

Amalgam is a condensable material with the unique property of marginal auto-sealing by oxide deposition with aging. The application of amalgam in the gingival part of the proximal cavity complemented by composite resin (amalcomp technique) provided a significant improvement in marginal seal compared to light-cured composite restorations^6^.

The polymerization shrinkage stress has been considered one of the main factors responsible for the marginal adaptation and microleakage of composite resin restorations^7^. This stress relies on the monomer composition of the composite, and might be controlled by modulations in the light activation process, which reduces the speed of the composite polymerization. Therefore, the control of initial light irradiance has been associated to the quality of the marginal seal in composite restorations^13^. Based on that, alternative light curing techniques, such as the soft-start activation, have been advocated aiming to reduce the shrinkage stress, with the same degree of conversion^13^, which could result in better marginal seal.

The aim of this study was to evaluate the gingival marginal seal and adaptation in class II composite restorations using different restorative techniques. The tested null hypothesis was that all restorative techniques produce similar performances.

## MATERIAL AND METHODS

Specimen Selection and Cavity Preparation

Thirty-two recently extracted human third molars were stored in saline at room temperature until use. The research protocol was approved by the School of Medicine’s Research Ethics Committee (018/2003 - UFPel, Brazil).

Standard Class II slot cavities were prepared in both mesial and distal surfaces using #1090 diamond burs (KG Sorensen, Barueri, SP, Brazil) mounted in a water-cooled high-speed turbine. The buccolingual extension of the cavities was 3 mm. Axial walls were prepared to a standard depth of 1 mm in dentin from the dentinoenamel junction. The gingival wall was located approximately 1.0 mm short of the cementoenamel junction (CEJ) in the mesial face (n=32) and 1.0 mm above CEJ in the distal face (n=32). The internal angles were rounded and cavosurface margins were finished with gingival margin trimmers^6^.

### Restorative Procedures

The prepared teeth were mounted between two dummy teeth using silicone (OK??) impression putty to reproduce proximal contact. An individual metal matrix was prepared for each tooth and stabilized with wooden wedges.

Teeth were randomly divided into 4 groups (n=8) and were restored as follows:

Group 1 (control): Composite resin/ conventional light curing: 35% phosphoric acid etching was done for 20 s followed by water rinsing for 30 s, and excess water was removed from the dentin surface with absorbent paper. Two consecutive coats of Single Bond adhesive system (3M/ESPE, St Paul, MN, USA; batch no. 1FB) were applied onto the cavity walls and light cured for 10 s with a halogen light source (Ultralux; Dabi Atlante, Ribeirão Preto, SP, Brazil; light irradiance = 450 mW/cm^2^). Filtek Z-250 composite resin (3M/ESPE; shade B2; batch no. 2MX) was inserted in 2 mm-thick oblique increments and light cured for 40 s.

Group 2: Composite resin/soft-start technique: the restorative procedures were similar to Group 1. However, in this group, the increments were light cured initially from a distance of 10 mm from the occlusal surface (determined by a periodontal probe) during 20 s followed by a 40-s curing time with the light guide tip contacting the occlusal surface. The average distance from the gingival wall to the occlusal surface was 7.96 mm in the dentin/cementum wall and 5.42 mm in the enamel wall.

Group 3: Amalcomp: the adhesive procedures were the same as above described. However, A 2-mm thick layer of amalgam (Logic Plus, batch no. 000250301, SDI, São Paulo, SP, Brazil) was condensed in the cervical region and allowed to set for 5 min. Two adhesive coats were applied to the amalgam and light cured for 10 s. Composite resin increments were inserted and light cured for 40 s.

Group 4: Open sandwich technique: Vitrebond RMGIC (Batch no. 2CY, 3M/ESPE) was prepared according to manufacturer’s instructions and a 2mm thick layer of material was injected into the cavity using a Centrix syringe and was light cured for 40 s. The cavity walls and the RMGIC surface were etched for 20 s, washed and dried as for Group 1. The adhesive system and the composite resin were used as previously described.

After 7 days of storage in distilled water, the teeth were removed from the silicone and the restorations were finished and polished using 30blade carbide burs and polishing disks (Sof Lex^TM^; 3M/ESPE) with diamond paste (FGM, Joinville, SC, Brazil).

### Microleakage Test and Evaluation

The teeth were thermocycled using 500 cycles from 5ºC to 55ºC with a dwell time of 30 s. The apex of each tooth was sealed with epoxy resin and the entire tooth surface was covered with two coats of nail varnish, except for the restorations and 1 mm around their margins. The specimens were immersed in 0.5% basic fuchsin solution for 24 h, followed by tap water washing for the same time. The specimens were embedded in acrylic resin and mounted in a low-speed, automatic precision cutting machine (Minitom, Struers, Copenhagen, Denmark). Three 1-mm thick mesiodistal slices were obtained per tooth using a low-speed diamond wheel saw (Sultrade; Com. Exp. Ltda, São Paulo, SP, Brazil) under water-cooling. The slices were examined using a stereomicroscope adapted to a digital camera. Each slab was scanned along with a millimeter scale and the digitized images were transferred to Image Tool software (San Antonio Dental School, University of Texas Health Science, TX, USA) in order to measure the length of dye penetration (in mm) along the gingival wall. Only the slice presenting the highest degree of penetration in each specimen was considered and recorded.

The microleakage data were analyzed statistically using non parametric Kruskal-Wallis and Mann-Whitney tests at 5% significance level.

### Qualitative Analysis of Marginal Adaptation

Three specimens from each group were randomly selected. Impressions (Express; 3M/ ESPE - batch no. 0GLY2C6) were taken of the tooth/restoration margins and replicas were obtained in epoxy resin (EMBED 812 KIT; EMS, Hatfield, PA, USA – batch no. 14120). The replicas were sputter-coated with gold-palladium and observed in a scanning electron microscope (XL30, Phillips International Inc., Potomac, MD, USA) on secondary electron image mode. Marginal adaptation was qualitatively evaluated observing the presence of gaps and voids at the tooth/ material interface (×200 magnification).

## RESULTS

Microleakage results for different groups in enamel and dentin/cementum margins are shown in Table 1.

**Table 1:** Microleakage mean values (in mm) and standard deviation (SD) for the different groups in enamel and dentin/ cementum margins

**Groups**	**Enamel margin Mean ± SD (mm)**	**Dentin/cementum margin Mean ± SD (mm)**
G1	0.28 ± 0.42 b	0.31 ± 0.25 a
G2	0.00 ± 0.00 a	0.43 ± 0.32 a
G3	0.30 ± 0.25 b	0.68 ± 0.26 b
G4	0.42 ± 0.25 b	0.68 ± 0.22 b

Different letters indicate statistically significant differences between groups (p<0.05).

There was lower leakage in enamel margins (p=0.001), except for G1. The lowest (p<0.05) dye penetration occurred for G2 (soft-start technique) in enamel margins, with no significant differences among the other groups.

In dentin/cementum margins, higher degree of leakage was observed for G3 and G4 compared to G1 and G2 (p<0.05), which were similar.

**Figure 1:**
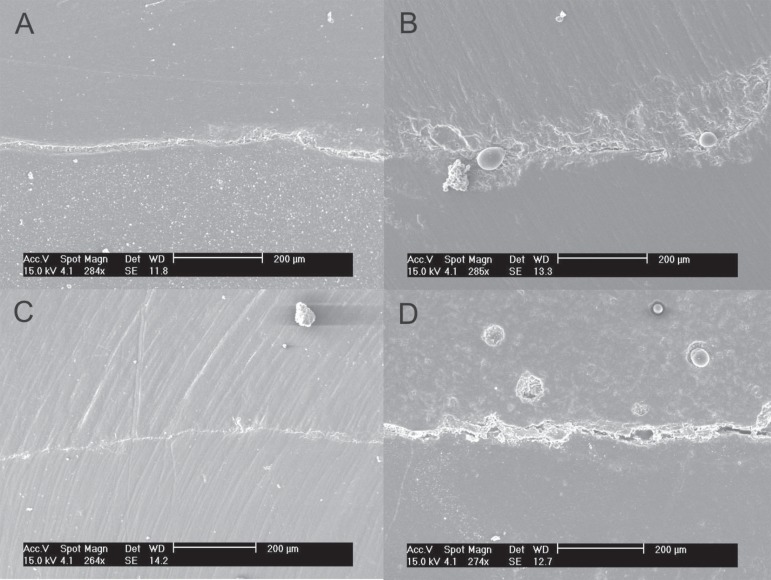
Marginal adaptation in enamel margins: A – G1 (Composite resin/conventional light-curing technique): a thin marginal gap was observed between the enamel and the composite restoration throughout the interface, similar to a superficial crack. B – G2 (Composite resin/soft-start technique): a very rare localized small marginal disruption observed at the tooth/restoration interface. C – G3 (Amalcomp): An adequate marginal adaptation between amalgam and the tooth was observed. D – G4 (Open sandwich technique): a wider gap was present throughout the whole interface and cohesive failure of the RMGIC was observed as rests of the restorative material remained adhered to the tooth structure

The qualitative analysis of the marginal adaptation in enamel is shown in Figure 1. For the control group, a thin marginal gap was observed between the tooth enamel and the composite restoration throughout the interface, similar to a superficial crack (Figure 1A). In the soft-start technique, a small marginal disruption was a localized feature rarely found in the tooth/ restoration interface (Figure 1B). Good adaptation between amalgam and tooth surface was observed in the amalcomp technique (Figure 1C). In contrast, for the open sandwich technique a wider gap was present throughout the interface with cohesive failure of the RMGIC (Figure 1D).

**Figure 2:**
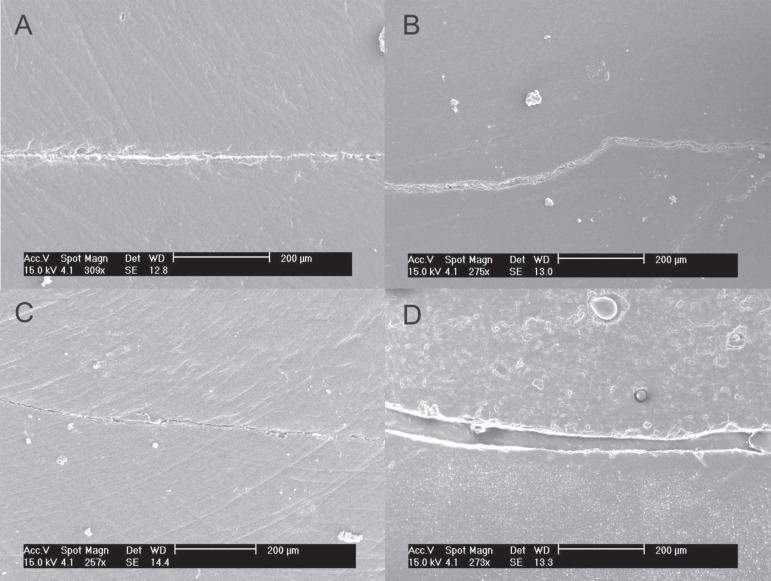
Marginal adaptation in dentin-cementum interface. A - G1 (Composite resin/conventional light-curing technique): marginal seal is apparently adequate similar to the image observed for this technique in enamel margins. B - G2 (Composite resin/soft-start technique): a thin gap was observed along the whole interface, which is a different feature found for this same technique in enamel margins (Figure 1B). C - G3 (Amalcomp): a thin gap was found throughout the amalgam-dentin interface. D - G4 (Open sandwich technique): a wide gap was found throughout the interface with no material adhered to the tooth structure, different from the findings in enamel margins

Representative images of the dentin-cementum marginal adaptation of restorations are shown in Figure 2. The control group exhibited an apparently adequate marginal adaptation, with a thin gap, which resembles that observed for the same technique in enamel. When the soft-start technique was evaluated (Figure 2B), a thin marginal gap was observed along the interface. In the amalcomp technique, a gap was found throughout the interface (Figure 2C), which is different from the feature observed for the same technique in enamel margins. The dentinrestoration interface for the open sandwich technique showed a wide gap throughout the interface with no material adhered to the tooth structure (Figure 2D), different from the findings in enamel margins (Figure 1D).

## DISCUSSION

Microleakage tests have been widely employed to screen the seal efficiency of restorations. Such tests face the challenge of reproducing the oral dynamics in an *in vitro* assay. Probably, the biggest limiting factor is the huge variability of methods, with no standardization, which impairs the comparison of the results^15^. In addition, results tend to present high variability, which must be taken into account when interpreting the statistical analysis. In spite of these limiting aspects, microleakage was chosen in this study because of its long-term report in literature. Furthermore, the test was designed taking into consideration the most frequent choices in test variables, as reported by Raskin, et al.^15^ (2001) in a systematic literature review.

The boundary conditions are fundamental to create the necessary bond strength to withstand the shrinkage stress^9^ and to direct the shrinkage vectors toward the cavity walls^18^. Several factors can be considered as potentially interfering in the adhesive process, namely the cavity depth, location, condition of the tissue and wetness. Enamel is basically an inorganic tissue and, therefore, a more stable substrate for adhesion, promoting a better marginal seal than dentin, as observed in the present study.

The soft-start technique (G2) produced no microleakage in enamel. In dentin/cementum margins it (G2) produced a marginal seal similar to the conventional technique (G1), which were lower than the other techniques (G3 and G4). This technique (G2) is based on the retard of the polymerization shrinkage by reducing the initial light irradiance^13,19^. According to Lim, et al. ^10^ (2002), the result of this delay is a longer time for the rearrangement of the composite molecules and for the stress release, which maintain adhesive links without ruptures^17^. Thus, the conversion rate during this initial period is lower compared to the full light irradiance. Nevertheless, it needs to be compensated by an increase in curing time^13^.

A 10-mm distance was used to reduce light irradiance in the first 20 s of polymerization. Yet, the real distance to the first composite increment in both, enamel and dentin, was higher than 10 mm (about 15 mm in enamel and 18 mm in dentin). Even though no measure of light irradiance was performed, one could infer a reduction of light irradiance based on Mehl, et al. ^13^ (1997), who observed a 50% decrease in irradiance working with a 10mm distance. Between 10 and 20 mm, the reduction of light irradiance was around 50 and 37% respectively, and was considered by the authors a good initial irradiance for improvement of the restoration seal. Failure in producing better seal in dentin margins might be explained by the difficulty of obtaining a good adhesion with such complex substrate, and is in accordance with previous reports^3,16^.

The application of amalgam in the gingival floor of proximal boxes has been related to good marginal seal^6^. It allows the use of the metallic matrix/wooden wedge that makes easier the reproduction of the proximal contact and the cervical adaptation. Unlike the composite resin, amalgam does not create pulling forces from the cavity and its condensation force is considered the most important factor in its marginal adaptation^1^. In fact, when observed under SEM, the amalgam produced an adequate adaptation to the enamel and a good adaptation to dentin. The advantageous auto-sealing is time dependent and relies on the deposition of oxides^20^. To avoid the occurrence of early microleakage on amalgam restorations a liner such as copal varnish is indicated^1^. Previous studies observed a better marginal seal in amalgam restorations when the cavity varnish was substituted by an adhesive system^4^. In this study, a single-bottle etch-andrinse adhesive system was used with the amalgam/composite restorations. It was light cured before the insertion of the amalgam, what may have avoided the micromechanical adhering of both materials and have caused the leakage reported in the study. Demarco, et al. ^6^ (2001) obtained the best sealing results with the amalcomp technique using a dual cure adhesive system (SBMPP) that probably created an intimate mechanical adherence with the amalgam, which was not the case in this study. In fact, when compared to the conventional technique, the amalcomp technique exhibited similar performance in enamel and worse marginal seal in dentin margins. Yet, the amalcomp technique represents a more sensitive and time-consuming technique, which could reduce its clinical applicability^6^.

In this study, the open-sandwich technique was not able to provide a better sealing than the other techniques. Fritz, et al.^8^ (1996) suggested dentin hybridization of dentin with adhesive system before RMGIC application to warrantee a dentinal tubule sealing in case of failure at the interface. In the present study, RMGIC was used without adhesive bonding agent, following the manufacturer indications for the material used. SEM evaluation showed cohesive failure of the RMGIC (Figures 2D and 3). Due to the powder/ liquid cement nature, these materials are very fragile. Apparently, the addition of the resinous content did not improve sufficiently the strength of the material to tensile loads. It seems that its brittleness did not allow it to withstand the shrinkage forces during the composite polymerization. Also, like amalcomp technique, the open sandwich technique is also a more sensitive and time-consuming procedure, which should be taken into account when proposing a restorative technique.

The null hypothesis was rejected since differences were observed between different techniques in enamel and dentin.

## CONCLUSIONS

Within the limitation of this *in vitro* study it can be suggest that: 1. The soft-start technique produced no microleakage in enamel margins; 2. None of the examined restorative techniques totally prevented dye penetration in dentin margins; 3. Marginal adaptation varied in both substrates and from different restorative techniques used.
